# Joint Image Processing with Learning-Driven Data Representation and Model Behavior for Non-Intrusive Anemia Diagnosis in Pediatric Patients

**DOI:** 10.3390/jimaging10100245

**Published:** 2024-10-02

**Authors:** Tarek Berghout

**Affiliations:** Laboratory of Automation and Manufacturing Engineering, Department of Industrial Engineering, Batna 2 University, Batna 05000, Algeria; t.berghout@univ-batna2.dz

**Keywords:** anemia diagnosis, conjunctival eye images, deep learning, fingernail images, LSTM network, model behavior, non-intrusive diagnosis, palmar images, pediatric patients, recurrent expansion

## Abstract

Anemia diagnosis is crucial for pediatric patients due to its impact on growth and development. Traditional methods, like blood tests, are effective but pose challenges, such as discomfort, infection risk, and frequent monitoring difficulties, underscoring the need for non-intrusive diagnostic methods. In light of this, this study proposes a novel method that combines image processing with learning-driven data representation and model behavior for non-intrusive anemia diagnosis in pediatric patients. The contributions of this study are threefold. First, it uses an image-processing pipeline to extract 181 features from 13 categories, with a feature-selection process identifying the most crucial data for learning. Second, a deep multilayered network based on long short-term memory (LSTM) is utilized to train a model for classifying images into anemic and non-anemic cases, where hyperparameters are optimized using Bayesian approaches. Third, the trained LSTM model is integrated as a layer into a learning model developed based on recurrent expansion rules, forming a part of a new deep network called a recurrent expansion network (RexNet). RexNet is designed to learn data representations akin to traditional deep-learning methods while also understanding the interaction between dependent and independent variables. The proposed approach is applied to three public datasets, namely conjunctival eye images, palmar images, and fingernail images of children aged up to 6 years. RexNet achieves an overall evaluation of 99.83 ± 0.02% across all classification metrics, demonstrating significant improvements in diagnostic results and generalization compared to LSTM networks and existing methods. This highlights RexNet’s potential as a promising alternative to traditional blood-based methods for non-intrusive anemia diagnosis.

## 1. Introduction

Anemia, a condition characterized by a deficiency in red blood cells or hemoglobin, is particularly critical in pediatric patients due to its potential impact on growth, development, and overall health. The causes of anemia in children can vary widely and include nutritional deficiencies, chronic diseases, genetic disorders, and bone marrow disorders. Common nutritional deficiencies contributing to anemia include iron, vitamin B12, and folate deficiencies. Chronic conditions, such as kidney disease, inflammatory disorders, and certain infections, can also lead to anemia by interfering with the production or lifespan of red blood cells. Genetic disorders, like sickle cell anemia and thalassemia, are hereditary causes that impact hemoglobin production or structure [1,2,3,4,5,6,7]. The symptoms of anemia in children can range from mild to severe and may include fatigue, pallor, shortness of breath, dizziness, and irritability. In more severe cases, symptoms may also include growth retardation, delayed development, and an increased risk of infections. The presence of these symptoms necessitates prompt diagnosis and intervention to mitigate potential adverse effects on a child’s health and development [8,9,10]. Treatment for anemia depends on the underlying cause and severity of the condition. For nutritional anemia, dietary modifications and supplementation are typically recommended to address deficiencies. Iron supplements are commonly prescribed for iron-deficiency anemia, while vitamin B12 or folate supplements are used for deficiencies in these nutrients. In cases of chronic anemia related to underlying diseases, managing the primary condition is crucial. For genetic or chronic forms of anemia, treatments may include medications, blood transfusions, or more advanced interventions, such as bone marrow transplants [3,4,11,12,13,14,15]. Given the diverse causes and implications of anemia, it is essential to utilize accurate diagnostic methods to tailor treatment effectively. Non-invasive diagnostic approaches, such as those leveraging image processing and deep learning, offer a promising avenue for the early and reliable detection of anemia in pediatric patients [16]. While traditional blood tests are the gold standard for diagnosing anemia, this study emphasizes the potential of imaging techniques as a complementary approach, particularly in contexts where access to laboratory facilities is limited or frequent blood draws pose challenges. Imaging offers unique benefits, such as non-invasiveness and the ability to capture additional physiological information, which can enhance accessibility in underserved or resource-limited environments. This research aims to highlight how imaging can serve as an adjunct to conventional methods, addressing the need for innovative diagnostic solutions in global health contexts.

In this context, analyzing related works in this specific field is crucial for identifying advancements, research gaps, and the need for new contributions. This is particularly important, given that this study focuses on medical image analysis, including conjunctival eye images, fingernail images, and palmar images. To ensure comprehensive coverage, the relevant literature employing similar types of images will be reviewed. The analysis will address key criteria, such as preprocessing methods, learning models, and potential limitations regarding the generalizability and applicability of these approaches in real-world scenarios. For instance, in [17], the authors utilized palmar images from 710 participants across hospitals in West Africa. These images were processed and segmented to extract features for training and testing machine learning models. An ensemble-learning model incorporating artificial neural networks (ANN), support vector machines (SVM), naive bayes (NB), decision trees (DT), and random forests (RF), combined with techniques such as stacking, boosting, bagging, and voting, was developed. In a comparative analysis, the stacking ensemble model demonstrated the highest classification accuracy, achieving 99.73%. One possible limitation of this study is that, while the accuracy is generally high (see Tables 3 and 4 from [17]), there are often perturbations and instability in other metrics beyond classification accuracy. This observation is also reflected in the performance of individual algorithms (see Table 2 from [17]). This suggests that data imbalance might be contributing to a higher classification accuracy but may result in poorer performance in other evaluation metrics, such as precision, recall, and F1 score. These discrepancies indicate potential gaps in the model’s robustness and generalizability, highlighting the need for further investigation into data-balancing techniques and the overall stability of the models across various performance metrics. In [18], a similar team collected a comprehensive dataset of 710 individuals aged below 6 years from the same region. This dataset, based on conjunctival images, is annotated with hemoglobin (Hb) levels (g/dL) to facilitate an accurate anemia diagnosis. A joint deep neural network was developed to analyze the dataset for anemia classification and Hb level estimation. The experimental results demonstrate the effectiveness of this network for both tasks. However, consistent with findings in [17], the metrics presented in [18] (see Table 3 and Figure 5 from [18]) show lower performance rates, with classification accuracies ranging from 74% to 85% and area under curve (AUC) values between 0.74 and 0.83. These results highlight the ongoing challenge of making definitive clinical decisions regarding patient health based on this approach. Another study, referenced in [19], utilized the same palmar dataset images as presented in [17] to perform a comparative analysis of machine-learning models. Following similar preprocessing steps, the study developed various models using algorithms, including convolutional neural networks (CNN), k-nearest neighbors (k-NN), NB, SVM, and DT for classification. Figure 13 from [19] indicates a similar instability in the performance metrics, which is consistent with the issues observed in previous studies. An additional study detailed in [20] expanded on previous work by incorporating palmar and conjunctival datasets, as well as a new dataset of fingernail images, all collected from the same region using similar methodologies. This study employed a comparable machine-learning comparative analysis, and the results presented in Section 4 of [20] demonstrated improved performance. However, the receiver operator curves (ROC) curves did not achieve the expected levels of performance, indicating that, while improvements were noted, the results may not yet be optimal for clinical decision-making.

In response to these limitations in related works, which are also summarized in Table 1, there is a growing need for deeper data processing than that of previous works, which can provide more accurate and realistic clinical conclusions. In addition, advances in deep learning offer more promising solutions than traditional machine-learning models. Therefore, this study aims to address this need by integrating advanced image-processing techniques with learning-driven data representation and model behavior.

The contributions of this study are as follows:

- Image processing—the study employs a detailed image processing pipeline to extract 181 features from 13 categories for anemia detection. The images are resized, converted to grayscale, and normalized. Segmentation isolates regions of interest, with additional calculations on region properties, color space conversions (hue, saturation, value (HSV) and A (green–red), B (blue–yellow) (LAB)), and texture features (gray-level co-occurrence matrix (GLCM) and extracting texture features (LBP)). Color moments, histograms, and edge detection further aid in analyzing color and texture variations. These features collectively enhance the non-intrusive detection of anemia by providing a comprehensive assessment of visual cues;
- Advanced learning techniques—a deep multilayered network based on long short-term memory (LSTM) is used to classify images into anemic and non-anemic cases. The hyperparameters are optimized using Bayesian approaches to enhance model performance;
- Innovative model framework—the LSTM model is incorporated into a new learning framework involving a recurrent expansion layer [24], forming the recurrent expansion network (RexNet). RexNet is designed to learn both data representations and model behavior, improving the understanding of the optimal data features for an accurate diagnosis;
- Application and evaluation—the proposed method is applied to three public datasets, namely conjunctival eye images, palmar images, and fingernail images, as described in previous studies [21,22,23]. Unlike prior works that primarily focus on ROC curves and accuracy metrics, this study includes a comprehensive range of both visual and numerical evaluations throughout all stages, from data preprocessing to model assessment. It features bar charts, illustrating class proportions and feature importance, scatter plots of extracted and selected features, learning curves, confusion matrices, ROC curves, and various other metrics. Additionally, this work compares the proposed approach with LSTM and other related methods, highlighting the advantages and limitations of the approach.

This paper is organized as follows. Section 2 describes the datasets used, including their collection methodology and processing. Section 3 presents the proposed approach, RexNet, along with its detailed learning rules. Section 4 showcases and explains the results obtained, while also discussing the study’s limitations and advantages. Finally, Section 5 offers conclusions and outlines future directions.

## 2. Materials

In this section, we will explore the datasets used in this work, including their collection methodology and the processes undertaken by the original developers to prepare them for use. We will also detail our proposed methodology for processing these images, ensuring that they are ready for the training phase of the models under investigation. In a third subsection, we will present the results of data visualization and processing, drawing key conclusions about the accuracy of the processing itself, as well as the complexity and necessity of our proposed deep-learning approach. 

### 2.1. Original Data Description

**Figure 1:**
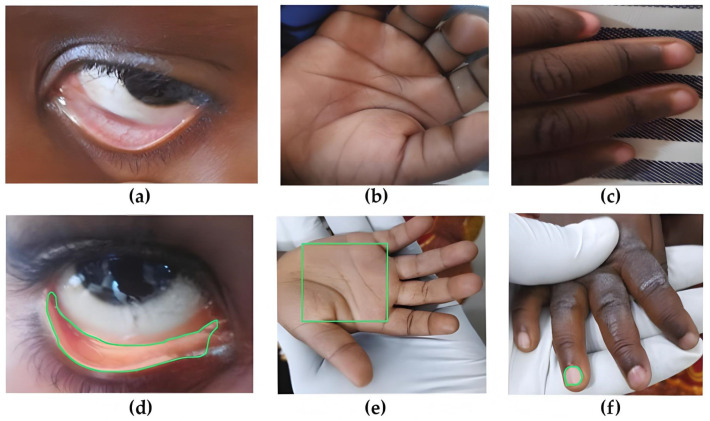
Example photographs of collected data and regions of interest. (**a**–**c**) Eye conjunctiva, hand palm, and fingernail images of non-anemic individuals; (**d**–**f**) eye conjunctiva, hand palm, and fingernail images of anemic individuals, with green zones highlighting the regions of interest in this study. Adapted from [20]: WILEY 2023, under open access license. The figure has been modified for better clarity, including realignment, recoloring of regions of interest, and denoising.

This study utilized three publicly available datasets [21,22,23] containing images of palpable palms, conjunctiva of the eyes, and fingernails. The dataset developers, focusing on data collection across multiple hospitals in West Africa, ensured that necessary preprocessing steps, including regions-of-interest (ROI) extraction and data augmentation, were performed before making the datasets publicly accessible. The data-collection system employed mobile tablets with a sufficient resolution of 12 megapixels and specific installed applications. Participant biodata, including Hb values, age, sex, and anemia status based on Hb levels, were recorded alongside the captured images and uploaded to cloud storage. Licensed biomedical scientists, trained for a week at the Centre for Research in Applied Biology, University of Energy and Natural Resources, Sunyani, Ghana, were responsible for capturing images of children aged under 5 years. The study was reviewed and approved by the Committee for Human Research and Ethics (Reference number: CHRE/CA/042/22) [20]. Conjunctiva images were captured by gently pulling down the lower eyelid, palm images by stretching the child’s hand, and fingernail images by holding the participant’s wrist. To avoid glare, which could compromise model performance, camera flashlights were turned off, minimizing the impact of ambient light. Data were collected from 10 health facilities, comprising seven district hospitals, two regional hospitals, and one tertiary hospital. The study focused on classifying participants as anemic (Hb < 11 g/dL) or non-anemic (Hb ≥ 11 g/dL). Across all datasets, a total of 710 photos were captured, with 424 images representing anemic individuals and 286 images representing non-anemic individuals. The images were processed using a triangle thresholding algorithm and augmented to increase the dataset size. ROI extraction involved segmenting the images based on distinct characteristics, with pixels assigned to one of the three CIELAB color space segments according to their intensity levels. The thresholding algorithm further divided the images into three classes, enabling precise ROI extraction [25]. After ROI extraction, image-augmentation techniques were applied to artificially expand the dataset. These included rotating the images by 90° and 270° and flipping them vertically and horizontally, all while maintaining the mean intensity values [26,27,28]. Additionally, the ROIs were translated by marginal shifts along image axes, preserving the mean intensity. Figure 1 presents examples of captured photos of the eye conjunctiva, hand palms, and fingernails for both anemic and non-anemic individuals. Visible symptoms, such as paleness in the conjunctiva, lighter coloration of the palms, and pale or brittle fingernails, are evident to the naked eye [29]. Additionally, ROIs, which were extracted by the dataset authors and made available for analysis, are highlighted in green to enhance understanding and provide a clearer focus on the areas that are crucial for the analysis and detection of anemia.

Table 2 presents an overview of the datasets collected from eye conjunctival images, summarizing key features such as hemoglobin level, age, severity of anemia, gender distribution, and hospital sources. Although specific details about the individual datasets are not available, it is important to note that all datasets were compiled by the same research team during closely aligned collection periods. This consistency in methodology and data gathering supports the idea that the findings related to one dataset can be generalized across the others. The mean HB level is reported at 10.35, with a standard deviation of 2.25, indicating a broad range in hemoglobin levels from a minimum of 3.1 to a maximum of 15. The average age of subjects is 31.58 months, with an age range of 6 to 60 months. In terms of severity classification, the dataset shows a distribution of mild (20.28%), moderate (32.68%), non-anemic (40.28%), and severe (6.76%). The gender distribution indicates a slightly higher proportion of males (56.90%) compared to females (43.10%). The data also includes contributions from various hospitals, with Ahmadiyya Muslim Hospital representing 18.03%, followed by Komfo Anokye Teaching Hospital at 18.87%, and other institutions contributing to the diversity of the sample.

### 2.2. Image-Preprocessing Methodology

**Figure 2:**
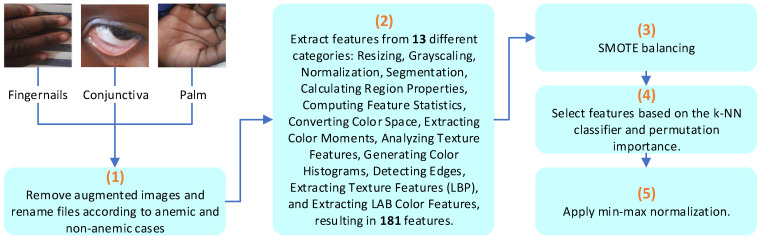
Flowchart of the proposed methodology for image processing.

In this work, the image-processing approach emphasizes the development of learning models rather than merely expanding the dataset through augmentation. As outlined in the flowchart of Figure 2, we prepared the three datasets [21,22,23] by first removing all augmented images and retaining only the original ones. Next, we ensured that the labels for all datasets were included in the filenames by renaming the original files. This step was essential to streamline the processing pipeline, allowing all images to pass through a unified workflow. Following this, we extracted 181 features across 13 different categories. These categories are resizing, grayscaling [30], normalization [31,32,33], segmentation [34,35], calculating region properties, computing feature statistics, converting color space, extracting color moments, analyzing texture features, generating color histograms, detecting edges, extracting texture features (LBP), and extracting LAB color features [36,37]. Subsequently, we conducted feature selection based on feature importance and performed a data-balancing process to generate synthetic data for minority classes.

As mentioned, the initial step involved cleaning the datasets by removing augmented images and retaining only the original ones. This ensured that the dataset contained only authentic samples for analysis. Additionally, filenames were modified to include relevant labels based on provided information and Excel sheets provided within datasets, facilitating a streamlined processing workflow. This preparation phase allowed for a unified approach to handling all images through a consistent pipeline. Next, feature extraction was a critical phase of the processing pipeline, involving a series of systematic preprocessing steps for each image. Initially, the images were resized to a uniform dimension of 128 × 128 pixels to ensure consistency in the input sizes for the subsequent processing steps. This standardization is essential for reliable feature extraction and analysis. Afterward, the images were converted to grayscale to simplify the data and then normalized to adjust the pixel values to have zero mean and unit variance [30]. This normalization process adjusts the image data to standardize the scale of pixel values across all images. Segmentation followed, where the normalized images were binarized using a global threshold determined by Otsu’s method [38]. This step isolates relevant regions of interest in the images by converting them into binary form based on the computed threshold. Subsequently, the segmented images were analyzed to extract the region properties, such as area, centroid, eccentricity, and solidity [38]. These properties provide essential information about the shapes and distributions of features within the segmented regions. In the next step, the images were converted to HSV and lightness using LAB color spaces to extract detailed color features [39,40]. Statistical moments, including the mean, standard deviation, and skewness for each color channel, were calculated to quantify the color characteristics of the images. For texture analysis, features were extracted using the GLCM and LBP [41,42,43]. GLCM features, such as contrast, correlation, energy, and homogeneity, were used to describe the texture of the images, while LBP features provided additional texture information based on local patterns. The equations that define these processes are described in (1)–(16). $I_{Norm}$ and $I_{Gray}$ are the normalized and gray-scaled images, respectively; μ and σ are the mean and standard deviations, respectively, of $I_{Gray}$. T is the threshold, and ${(\mu }_{G\cdot },\omega \left(t\right),\mu (t))$ are the weight of the class below the threshold t, the mean intensity of the class below the threshold t, and the global mean intensity of the $I_{Norm},$ respectively. The coordinates of pixels in image $I_{Norm}$ are $\left(a,b\right).\;A$ is the area of the region. $C_{a}$ and $C_{b}$ are centroids of a and b, respectively. E is the eccentricity. ${l_{a}}^{2}$ and ${l_{b}}^{2}$ are the squared lengths of the semi-major axis, the length of the semi-minor axis of the ellipse that has the same second moments as the region. S is solidity, and C is the area of the convex hull surrounding the region. $\mu_{c}$, $\delta_{c}$, and ${Skweness}_{c}$ are extracted color moments statistics. $P_{i}$ is the pixel value. LBP are local binary features, and np are the index of neighborhood pixels. Note that the summations in Equations (4)–(16) represent the number of pixels in the respective images and are omitted for clarity.

(1) $$I_{Norm}=\frac{I_{Gray}-\mu }{\sigma }$$

(2) $$T=argmax(\frac{[\mu_{G\cdot }\omega \left(t\right)-\mu (t){]}^{2}}{\omega \left(t\right)(1-\omega \left(t\right))})$$

(3) $$I_{Bin}(a,b)=\left\{\begin{array}{c} 1\;\mathrm{if}\;I_{Norm}(a,b)\geq T\\ 0\;\mathrm{if}\;I_{Norm}(a,b)<T \end{array}\right.$$

(4) $$A=\sum_{(a,b)\in R}I_{Bin}(a,b)$$

(5) $$C_{a}=\sum_{(a,b)\in R}{a\cdot I}_{Bin}(a,b)$$

(6) $$C_{b}=\sum_{(a,b)\in R}{b\cdot I}_{Bin}(a,b)$$

(7) $$E=\sqrt{\frac{{l_{a}}^{2}}{{l_{b}}^{2}}}$$

(8) $$S=\frac{A}{C}$$

(9) $$\mu_{c}=\frac{1}{N}\sum P_{i}$$

(10) $$\delta_{c}=\sqrt{\frac{1}{N}\sum \left(P_{i}-\mu_{c}\right)}$$

(11) $${Skweness}_{c}=\frac{1}{N{\delta_{c}}^{3}}\sum {\left(P_{i}-\mu_{c}\right)}^{3}$$

(12) $$Contrast=\sum \sum {{\left(a-b\right)}^{2}P}_{\left(a,b\right)}$$

(13) $$Correlation=\frac{\sum \sum (a-\mu_{a})(a-\mu_{b})P_{\left(a,b\right)}}{\delta_{a}\delta_{b}}$$

(14) $$Energy=\sum \sum {P_{\left(a,b\right)}}^{2}$$

(15) $$Homogeneity=\sum \sum \frac{P_{\left(a,b\right)}}{1+\left|a-b\right|}$$

(16) $$LBP=\sum sgn(I_{Bin\left(np\right)}-I_{Bin}(a,b))\cdot 2^{np}$$

Following feature extraction, the next step was to perform feature selection based on their relevance to the classification task. I used a K-nearest neighbors (K-NN) model with the number of neighbors set to 5 to evaluate the importance of each feature in distinguishing between the classes. The assumption behind using K-NN for this task is that features contributing more to reducing the classification error in this local neighborhood are considered more relevant. A threshold for feature importance was set, varying between 40% and 60%, depending on the dataset type, to ensure that only the most informative features were retained [44,45]. Specifically, features that significantly contributed to the K-NN’s classification accuracy were kept, while those below the threshold were discarded, reducing the original set of 181 features. This step helped streamline the data by focusing on the most meaningful features for the model. Finally, data normalization was performed to scale the retained features between 0 and 1, as shown in Equation (17), ensuring consistent input for model training. In this process, x represents the extracted features, and x′ refers to the normalized version of these features.

(17) $$x\prime =\frac{x-x_{min}}{x_{max}-x_{min}}$$

To address the class imbalance in the dataset, we employed the synthetic minority over-sampling technique (SMOTE), which is a method grounded in the KNN algorithm [46]. SMOTE generates synthetic samples for minority classes by identifying k-nearest neighbors using a default of five neighbors and creating new instances along the line segments that connect these neighbors. This approach enriches the representation of the minority class, allowing the model to learn more effectively from its feature space and enhancing the decision boundary between classes. Additionally, our processing pipeline included data cleaning to remove inconsistencies, feature extraction to highlight relevant attributes, and normalization to ensure proper scaling. This comprehensive preparation not only balanced the dataset but also facilitated robust analysis and model training, ultimately improving predictive performance and reliability.

### 2.3. Data Visualization and Preprocessing Results

**Figure 3:**
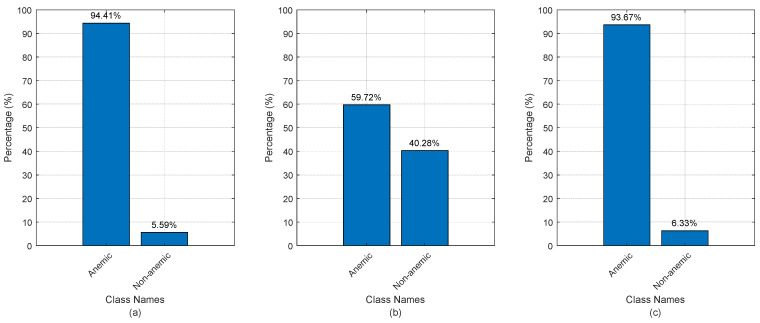
Class proportions of image datasets: (**a**) palmar images; (**b**) eye conjunctiva images; and (**c**) fingernail images.

Figure 3 illustrates the class proportions of the original data, highlighting a primary indication of data complexity and addressing limitations in previous works due to significant data imbalance [17,18,19,20]. Specifically, the number of non-anemic cases is notably smaller compared to anemic cases. This imbalance implies that the model may be biased towards predicting the majority class (anemic cases) and could struggle to accurately identify the minority class (non-anemic cases). Even if classification accuracy in previous works may appear to be high, the lower values in F1 score, recall, and precision reveal variability in results from one test to another, further underscoring the challenges posed by the imbalanced dataset [17,18,19,20]. This emphasizes the need for strategies to address class imbalance and improve the robustness of model performance. And this is the reason for including the SMOTE technique in this work. An exception is observed in the eye conjunctival images, where the class proportions are distributed almost equally. This balanced distribution provides a more realistic dataset for analysis, reducing the risk of bias towards any particular class and offering a more accurate reflection of the conditions being studied. This balanced class proportion in eye conjunctival images demonstrates a more straightforward scenario for classification, potentially leading to more reliable and consistent performance metrics compared to the imbalanced datasets for other image types.

**Figure 4:**
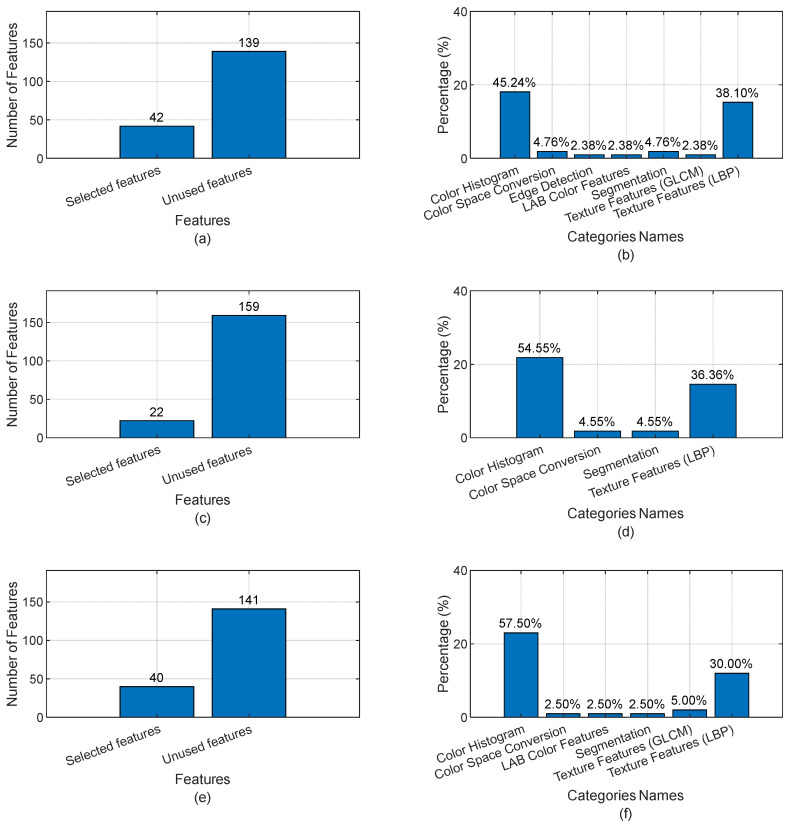
Selected feature categories and their proportions based on the feature-extraction process for each dataset. (**a**,**b**) Proportions of selected feature categories for the palm dataset; (**c**,**d**) proportions of selected feature categories for the eye conjunctiva dataset; and (**e**,**f**) proportions of selected feature categories for the Fingernails dataset.

Figure 4 provides an overview of the proportions and types of features utilized in this work. It details the proportions of features derived from each category across different datasets, as shown in the bar charts. These proportions vary from one dataset to another. However, a common conclusion is evident, namely that color histogram features and texture features, specifically LBP, are the most influential in the learning process. This is likely due to the nature of the datasets and images used, which emphasize color and texture information as critical for effective classification and analysis. Additionally, the analysis reveals that palm images and fingernail images require or showcase a greater variety of feature types compared to eye conjunctival images. This observation suggests that the complexity and variability in palm and fingernail images necessitate a broader set of features to capture the intricate details and patterns effectively. This might be attributed to the diverse structural and textural characteristics present in palm and fingernail images, which differ significantly from the more uniform features of eye conjunctival images. Thus, incorporating a wide range of features in the model improves its robustness and accuracy in distinguishing between different conditions based on these images.

**Figure 5:**
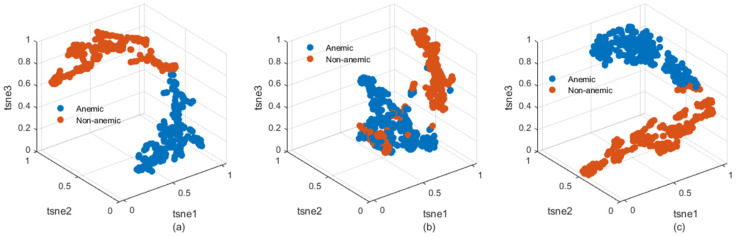
Dataset class scatters after image processing. (**a**) Palmar images; (**b**) eye conjunctiva images; and (**c**) fingernail images.

Figure 5 displays scatter plots generated using t-SNE (t-distributed stochastic neighbor embedding) for three datasets, showcasing the balanced size of samples and highlighting the effectiveness of the well-structured processing [47,48]. The 3D scatter plots reveal a clear agglomeration of samples belonging to similar classes, demonstrating that the feature extraction and processing methods have successfully organized the data according to class similarities. Additionally, the plots show a distinct separation between samples from different classes across all datasets, indicating the ability of the features to differentiate between various health conditions. This clear separability, as visualized through the t-SNE distributions, suggests an enhancement in anemia diagnosis, as the improved data-processing techniques contribute to more accurate and reliable classification of health cases. In the dataset scatter plots observed in Figure 5b, an additional observation regarding the eye conjunctival image dataset can be noted. Given that the data are almost balanced in their raw form (as shown in Figure 3b), some samples from different classes exhibit similar patterns. This introduces a level of complexity but reflects a more realistic scenario, allowing for more nuanced conclusions to be drawn during the learning process. This complexity underscores the importance of using representation-learning algorithms, such as deep learning, to capture and differentiate subtle patterns effectively. In this context, the use of advanced architectures like RexNet, as suggested in this work, is particularly valuable for improving classification performance and handling such nuanced data distributions.

## 3. Methods

As previously mentioned, this work adopts RexNet, which builds upon both the LSTM network and recurrent expansion rules [24,49]. While LSTM networks are traditionally designed for time-series data, they were chosen here due to their ability to capture long-term dependencies and complex patterns. In this study, the image data are first preprocessed to extract critical features, such as texture and shape, transforming them into sequences that the LSTM can efficiently process. Each feature vector is treated as a step in a sequence, allowing the LSTM to learn relationships between different aspects of the image. This approach enables the model to capture intricate patterns and correlations within the features extracted from the images, enhancing the diagnosis of anemia in young children. Furthermore, by integrating these extracted image features with other factors, such as age, the model leverages LSTM’s strength in managing sequential data, providing a richer, more accurate diagnostic capability. This approach allows RexNet to capture the important patterns and dependencies between different features, leading to more accurate and robust diagnostic outcomes. Inspired by the concept of recurrent expansion, which involves recursively collecting inputs, mappings, and estimated targets (IMTs) from several learning models and feeding them into subsequent networks, as discussed in (18). RexNet facilitates the integration of an entire pretrained network as a layer within new deep networks, as described in (19). $x_{k+1}$ represents the IMTs mapped by the previously trained deep network, and x represents the inputs of our dataset. φ(x) refers to the hidden LSTM layer, and $d(\varphi \left(x\right))$ are the features obtained from the dropout layer. $\tilde{y}$ are estimated outputs, and n stands for the number of recurrent networks. And ($\rho_{1},\rho_{2},\rho_{3}$) represent user-specified processing functions or algorithms designed to reduce the complexity of mapping and processing estimated targets. This is particularly important given the potential size of the mappings (hidden layers) and the need for additional operations, such as rescaling, to ensure accurate and efficient processing. In the Rex layer, there is no need to incorporate these functions, as dropout and activation functions inherently manage this stage. These components will also be fine-tuned in conjunction with the learning algorithm itself.

(18) $$x_{k+1}=[x,\rho_{1}(\varphi (x{)}_{i=1}^{n}),\rho_{2}(d(\varphi \left(x\right){)}_{i=1}^{n}),\rho_{3}((\tilde{y}{)}_{i=1}^{n})]$$

(19) $$x_{k+1}=[x,\varphi \left(x\right),d(\varphi \left(x\right)),\tilde{y}]$$

**Figure 6:**
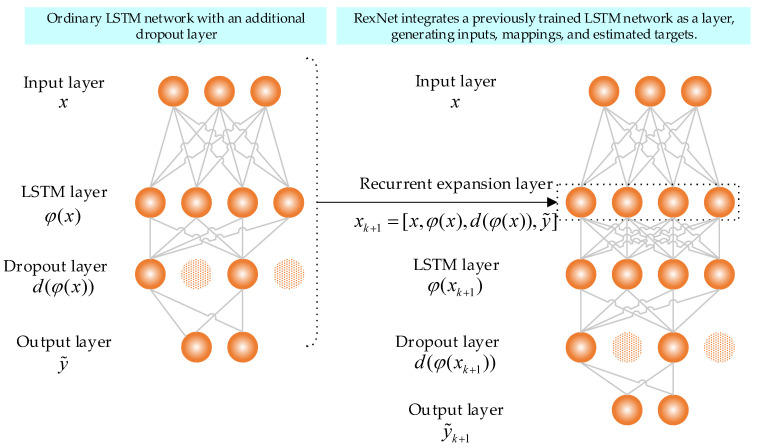
Flow diagram of proposed RexNet.

The RexNet approach offers a more efficient alternative to training a complete deep network with IMTs, which can be highly complex. Instead, it fine-tunes a new layer in the deep network while retaining the previous network solely for mapping purposes. This process is illustrated in Figure 6 with a simplified diagram. In this figure, a dropout layer is incorporated to reduce computational costs, which can arise from the large size of the IMTs. In this work, the concept of understanding “model behavior” refers to ensuring that a specific model (e.g., RexNet) can enhance the learning process more effectively than traditional deep-learning models (e.g., LSTM), when the interaction between dependent and independent variables is known through a specific mapping (e.g., IMTs). Unlike LSTM, which primarily relies upon inputs as the sole source for training, RexNet draws from a variety of information sources. This approach is particularly beneficial when analyzing responses from a deep network, as it allows RexNet to gain insights into the model’s behavior and optimize the learning process accordingly [50].

In addition to learning the rules of RexNet presented in Equations (18) and (19), the LSTM unit includes an input gate $i_{t}$, a forget gate $f_{t}$, a cell state $C_{t}$, and an output gate $o_{t}$ [49]. These gates and cells regulate the amount of new input to allow in, determine what information from the previous cell state to discard, store long-term data, and control how much of the cell state to output. They are determined according to (20)–(25), where $x_{t}$ is the input at the time step t, $h_{t-1}$ is the hidden state from the previous time step, δ is the sigmoid function, tanh is the hyperbolic tangent function, W and U are weight matrices, b is the bias vector, and ⊙ denotes element-wise multiplication.

(20) $$i_{t}=\sigma \left(W_{i}x_{t}+U_{i}h_{\left\{t-1\right\}}+b_{i}\right)$$

(21) $$f_{t}=\sigma \left(W_{f}x_{t}+U_{f}h_{t-1}+b_{f}\right)$$

(22) $$o_{t}=\sigma \left(W_{o}x_{t}+U_{o}h_{t-1}+b_{o}\right)$$

(23) $${\tilde{C}}_{t}=tanh\left(W_{c}x_{t}+U_{c}h_{t-1}+b_{c}\right)$$

(24) $$C_{t}=f_{t}⊙C_{t-1}+i_{t}⊙{\tilde{C}}_{t}$$

(25) $$h_{t}=o_{t}⊙tanh\left(C_{t}\right)$$

Bayesian optimization was selected for fine-tuning the hyperparameters of both the LSTM and RexNet models due to its capability to efficiently handle large parameter spaces and deliver fast, accurate optimization results [51,52]. This method was utilized to optimize a total of 11 hyperparameters, including the number of hidden units, maximum epochs, mini-batch size, initial learning rate, gradient threshold, L2 regularization, dropout rate, recurrent dropout rate, state activation function, gate activation function, and sequence length. Each parameter was adjusted within the specified ranges, as outlined in Table 3. The choice of Bayesian optimization was driven by its effectiveness in navigating complex parameter spaces and achieving high-quality results with fewer evaluations, making it well-suited for optimizing models with extensive hyperparameter configurations [52].

## 4. Results

In this section, we will present the results from the trained RexNet and LSTM models, followed by a comparison with previous work in the field of anemia diagnosis. We will also address the limitations and potential gaps in our approach, aiming to guide future research efforts toward possible improvements. The results will be illustrated through key visual metrics that highlight convergence and learning behavior. This will be followed by a numerical evaluation presented in the tables. Each result will be discussed in detail to provide a comprehensive understanding of the models’ performances and the effectiveness of our methodology.

### 4.1. Results and Comparison 

**Figure 7:**
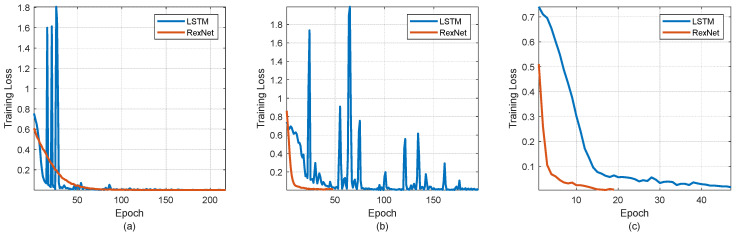
Training behavior of the studied approaches. (**a**) Loss function of RexNet and LSTM for the fingernails dataset; (**b**) loss function of RexNet and LSTM for the eye conjunctiva dataset; and (**c**) loss function of RexNet and LSTM for the palmar images dataset.

The results of the loss function training, as illustrated in Figure 7, highlight the performance of both LSTM and RexNet models across different datasets. The figure provides a comparative analysis of the training loss for both models on three datasets, namely fingernails, eye conjunctiva, and palmar Images. The loss function is a critical measure of how well the model is learning; a lower loss indicates better performance. In particular, the results show that RexNet exhibits a faster and smoother convergence compared to LSTM across all datasets. This observation supports previous theories regarding the efficiency of RexNet in learning from data representations and model behavior. The quicker convergence of RexNet suggests that it is more effective at minimizing the loss function during training, which implies a more efficient learning process. The smoother convergence curve of RexNet also indicates greater stability and consistency in training. This behavior aligns with the understanding that RexNet’s architecture and training mechanisms are well-suited for capturing and learning complex patterns in data. Overall, the comparative analysis in Figure 7 underscores the advantages of RexNet in terms of training efficiency and stability, reinforcing the theoretical insights into model behavior discussed earlier.

**Figure 8:**
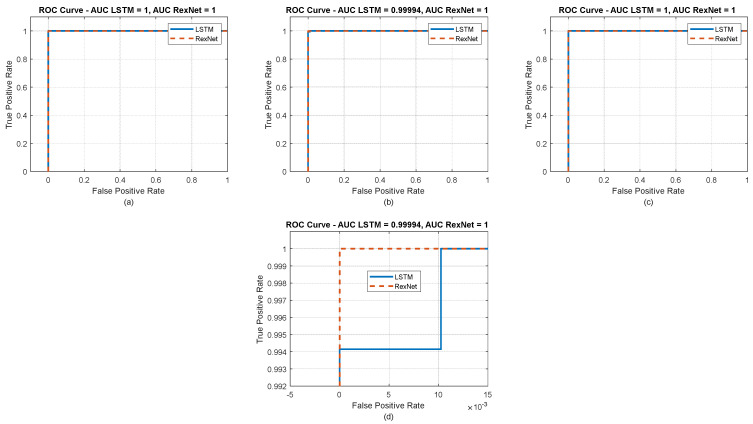
ROC curves behavior of the studied approaches. (**a**) ROC curves of RexNet and LSTM for the fingernails dataset; (**b**) ROC curves of RexNet and LSTM for the eye conjunctiva dataset; (**c**) ROC curves of RexNet and LSTM for the palmar images dataset; and (**d**) zoomed-in subplot for dataset 2—palm dataset.

The ROC curves displayed in Figure 8 illustrate a clear performance comparison between the LSTM and RexNet models across three different datasets. The curves demonstrate that RexNet consistently maintains higher or equal true-positive rates (TPR) compared to LSTM at various false-positive rates (FPR), indicating a generally better performance in correctly identifying positive cases. The AUC values, shown at the top of each subplot, further support this observation by revealing that RexNet often achieves slightly higher AUC values than LSTM, with some cases where the values are equal. This suggests that RexNet not only matches but sometimes surpasses LSTM in overall classification performance, effectively balancing TPR and FPR. The higher AUC values for RexNet imply a more robust performance in distinguishing between classes, even though both models exhibit strong performance. This comparison highlights RexNet’s advantage in terms of overall effectiveness while still recognizing the competitive performance of LSTM across the datasets.

**Figure 9:**
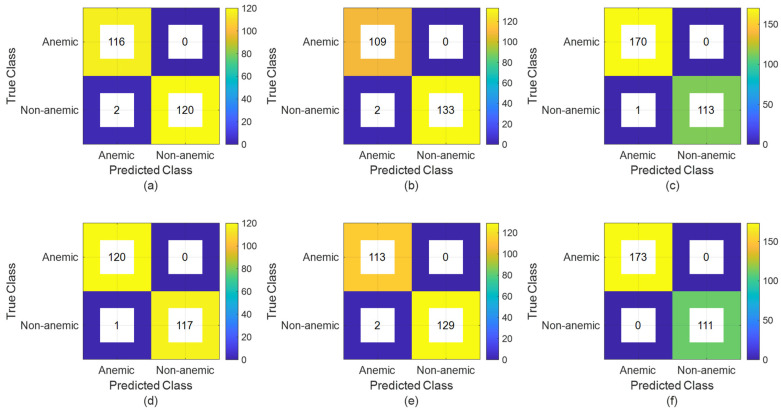
Confusion matrices for LSTM and RexNet models across different datasets. (**a**–**c**) LSTM results on fingernails, palm, and conjunctival eye datasets; (**d**–**f**) RexNet results for fingernails, palm, and conjunctival eye datasets.

The darker blue areas in the confusion matrices for false positives and false negatives in Figure 9 signify that RexNet’s prediction errors are generally fewer than or equal to those of LSTM. This observation is further supported by the numerical values, which show lower counts of misclassifications in RexNet compared to LSTM. Specifically, the numbers associated with RexNet exhibit a notable reduction in false positives and false negatives across the datasets, indicating better accuracy. The pronounced dark blue regions in RexNet’s confusion matrices, along with the corresponding numerical evidence, highlight its superior ability to classify both “Anemic” and “Non-anemic” categories, ultimately reflecting improved overall performance and lower error rates.

The comparison of the studied approaches in Table 4, particularly between the RexNet and the LSTM models, reveals that RexNet consistently outperforms the existing methods in terms of accuracy, precision, recall, and F1 score across multiple datasets, such as eye, fingernails, and palm. For instance, while both LSTM and RexNet show excellent performance on the eye dataset during training, RexNet achieves perfect scores across all metrics during testing, something that LSTM does not. RexNet’s generalizability is further underscored by its higher precision and recall values, particularly in scenarios where LSTM’s performance drops, such as with the fingernails dataset during testing. When compared to the existing work cited in [19,20], RexNet shows a substantial improvement. For example, for the eye dataset, the work in [20] reports a significantly lower accuracy and recall compared to RexNet’s results, indicating that RexNet is more reliable for this particular application. Similarly, the LSTM model shows a drop in performance on the fingernails dataset during testing, whereas RexNet maintains high accuracy and generalizes better to unseen data. This discrepancy suggests that previous studies might not have fully optimized their models or data-preprocessing techniques, which could explain the lower performance. It is important to note that, while RexNet demonstrates superior performance, its longer training times, especially on the fingernails dataset, indicate a trade-off between accuracy and computational efficiency. For real-world applications where time is a critical factor, this could be a limitation. However, for scenarios where accuracy is paramount, RexNet would be the recommended model. Given these findings, the recommendation is to consider RexNet for applications requiring high accuracy and robust performance across different datasets. However, if computational resources or time efficiency are constraints, LSTM could be a viable alternative, though with slightly reduced generalization capabilities. For future work, exploring optimization techniques to reduce RexNet’s training time without compromising its superior performance would be beneficial. Additionally, further analysis of the discrepancies observed in the existing works might provide insights into improving generalization across different models and datasets.

Lastly, Table 5 presents a comparison of the hyperparameter-tuning results for RexNet and LSTM across three datasets, namely the palmar images, eye conjunctival, and fingernails. The results highlight RexNet’s advantages in terms of computational efficiency and flexibility in hyperparameter selection. For instance, RexNet consistently requires fewer hidden units (e.g., 61 vs. 185 for the palmar images) and fewer epochs to converge (e.g., 19 vs. 47 for the palmar images), demonstrating its ability to achieve similar or better performance with reduced computational resources. Additionally, RexNet’s flexibility in exploring various activation functions, such as soft sign and hard sigmoid, contrasts with LSTM’s reliance on standard functions like tanh and sigmoid. This flexibility allows RexNet to better capture complex interactions in the data, which is particularly valuable for tasks like anemia diagnosis. Moreover, RexNet’s custom layer design, implemented as a class function in MATLAB, further enhances computational efficiency. MATLAB’s binary encoding accelerates the execution of these custom layers, making RexNet a powerful and resource-efficient alternative to traditional LSTM networks. Overall, the table underscores RexNet’s superior performance and flexibility, making it an excellent choice for complex modeling tasks, where both speed and accuracy are crucial.

Table 6 presents an analysis of network sizes, highlighting the trainable parameters of both LSTM and RexNet across the three medical imaging datasets used for anemia diagnosis. For the Eye dataset, RexNet exhibits a significant number of parameters (21,802) compared to LSTM (5698), indicating its capacity to capture complex features in medical images. Conversely, in the fingernails dataset, LSTM has more trainable weights (29,642) than RexNet (12,451), suggesting it may be better suited for that specific dataset. The palm dataset follows a similar trend, with LSTM again showing a higher count of parameters (42,552) than RexNet (17,875). Overall, RexNet’s ability to extract intricate features, coupled with its generally lower parameter count in two datasets, makes it an attractive option for medical image analysis. Therefore, it is recommended to prioritize RexNet for anemia diagnosis, especially in contexts where image complexity is high, as it can enhance diagnostic accuracy while potentially reducing training time and mitigating the risk of overfitting.

### 4.2. Overal Discussion, Comparisons, and Limitations

This study was conducted using original datasets, without the inclusion of augmented images. The initial results indicated that the data were highly imbalanced, with varying ratios across different datasets (refer to Figure 3). This imbalance does not fully align with the dataset descriptions provided in previous works (e.g., [18,19,20]). It is worth noting that prior studies primarily relied on limited data-processing techniques and did not adequately address the issue of data imbalance. While data augmentation was employed in earlier works, it was used primarily to increase the dataset size rather than to specifically target and correct the imbalance. Additionally, previous studies reported higher performance metrics in their numerical results. However, visual representations, such as bar charts, ROC curves, and AUC areas sometimes indicated lower performance, potentially leading to contradictions between the reported numerical data and visual outcomes. In contrast, our work involved more comprehensive data preprocessing, which significantly enhanced data scatter plots and improved the separability of data classes. Furthermore, we employed a wide range of visual metrics to validate the accuracy and reliability of our results. These, combined with rigorous numerical evaluations, contribute to a more robust analysis, reinforcing the credibility and effectiveness of our approach. Therefore, comparing our work directly with existing methods, as if they are equivalent, is not entirely fair due to the different levels of data preprocessing and the more thorough approach taken in our study. The depth of data preprocessing and the focus on addressing data imbalance in our work set it apart from previous methods, leading to potentially more reliable and accurate results.

In the case of the fingernails and palmar image datasets, the number of non-anemic samples is significantly smaller compared to the anemic cases (refer to Figure 3). Although the SMOTE was used during data processing to address this imbalance, it is important to recognize that SMOTE, while effective, can sometimes lead to unrealistic conclusions when the minority class is very small [46]. This is because SMOTE generates synthetic samples by interpolating between existing minority class samples. When the minority class is too small, the generated samples may not capture the true variability and complexity of the minority class, leading to an overestimation of the model’s performance in this class [46]. This can create a false sense of confidence in the model’s ability to generalize to new, unseen data, particularly for the minority class. Thus, while SMOTE has its benefits for balancing the dataset, in scenarios where the minority class is extremely limited, as in these datasets, the conclusions drawn about model performance may need to be carefully considered. Additional strategies, such as gathering more data for the minority class or employing more sophisticated sampling techniques, might be necessary to ensure that the model’s performance is both realistic and reliable.

## 5. Conclusions

In conclusion, this study represents a significant advancement in non-intrusive anemia diagnosis for pediatric patients by integrating image processing with advanced learning models. By extracting 181 features from 13 categories, we ensured a comprehensive representation of the data, which proved critical for the learning models. The feature-selection process identified the key variables, including texture and histogram color features, that enhanced the learning performance and improved diagnostic accuracy. Our approach utilized LSTM, optimized through Bayesian hyperparameter tuning, and was subsequently integrated into the RexNet model. RexNet demonstrated superior performance across three public datasets, namely conjunctival eye images, palmar images, and fingernail images, highlighting its robustness in generalizing across different types of input data. Notably, RexNet outperformed traditional LSTM networks and the existing methods across various metrics, both visual and numerical, indicating its potential as a more reliable tool for anemia diagnosis. However, the study identified limitations, particularly for datasets where non-anemic samples were significantly smaller than anemic cases, such as in the fingernail and palmar image datasets. While the use of SMOTE helped address class imbalance, it may have led to unrealistic conclusions due to the small size of the minority class. This limitation underscores the need for careful consideration in data sampling and model evaluation to ensure reliable diagnostic outcomes. Overall, RexNet’s ability to achieve higher results across multiple metrics establishes it as a promising alternative to traditional blood-based diagnostic methods.

## Figures and Tables

**Table 1:** Summary of related works analysis results.

Ref.	Year	Image Processing	Dataset	Learning Tools	Advantages	Limitations
[17]	2023	Background Removal; Binary Image; Grayscale Conversion; Image Enhancement; Median Filter; Noise Reduction; Region-of-Interest (ROI) Extraction; RGB-to-YCbCr Conversion; Thresholding.	Palmar images [21].	ANN; Bagging; Boosting; DT; NB; RF; Stacking; SVM; Voting.	Higher classification accuracy.	Higher variability is observed in both the tables and visual evaluations (curves and charts) in these works, particularly in achieving stable F1 scores, recall, and precision. This issue is believed to stem primarily from the lack of discussion on data imbalance.
[18]	2023	A* Components; CIE L*a*b* Color Space (CIELAB); Color Characterization; Device-Independent Digital Representation; Image Extraction; Pearson Correlation Index; Red Components (a* > 0); ROI Conversion; Standard Deviation Value.	Eye conjunctival pallor [22].	VGG16; ResNet50; DenseNet121; Vision Transformer (ViT); ConvNeXtBase.		
[19]	2023	Augmented Dataset; CIE L*a*b* Color Space (CIELAB); Extracted Images; Flipping; Pre-processing; Region-of-Interest (ROI) Segmentation; Rotation; Translation.	Palmar images [21].	CNN; Decision Tree; k-NN; Naive Bayes; SVM.		
[20]	2023	Augmentation; CIE L*a*b* Color Space; Extraction; ROI Extraction; Segmentation; Triangle Thresholding Algorithm.	Palmar images [21]; Eye conjunctival pallor [22]; Fingernails image dataset [23].	NB; CNN; SVM; k-NN; DT.		

*A*, L*, and a* refer to specific components of the CIE L*a*b* (CIELAB) color space, where L* represents lightness, a* the red-green axis, and b* the blue-yellow axis. Positive a* values indicate red. The CIELAB space provides a device-independent color representation, ensuring consistency across different devices.

**Table 2:** Overview of datasets collected from eye conjunctival images.

Feature	Mean	Standard Deviation	Min	Max	Categories
Hemoglobin level	10.35	2.25	3.1	15	-
Age (months)	31.58	16.78	6	60	-
Severity	-	-	-	-	Mild (20.28%), Moderate (32.68%), Non-Anemic (40.28%), Severe (6.76%)
Gender	-	-	-	-	Female (43.10%), Male (56.90%)
Hospital	-	-	-	-	Ahmadiyya Muslim Hospital (18.03%), Bolgatanga Regional Hospital (13.38%), Ejusu Government Hospital (5.77%), Holy Family Hospital (1.13%), Kintampo Municipal Hospital (8.45%), Komfo Anokye Teaching Hospital (18.87%), Manhyia District Hospital (6.06%), Nkawie-Toase Government Hospital (12.11%), SDA Hospital (2.11%), Sunyani Municipal Hospital (14.08%)

**Table 3:** Training hyperparameters.

Hyperparameter	Range	Type
Number of hidden units	[10, 200]	Integer
Maximum epochs	[10, 1000]	Integer
Mini-batch size	[16, 250]	Integer
Initial learning rate	[0.0001, 0.1]	Real
Gradient threshold	[0.1, 1]	Real
L2 regularization	[0.0001, 0.1]	Real
Dropout rate	[0, 0.5]	Real
Recurrent dropout rate	[0, 0.5]	Real
State activation function	tanh–softsign	Categorical
Gate activation function	sigmoid–hard sigmoid	Categorical
Sequence length	[10, 100]	Integer

**Table 4:** Comparisons of studied approaches and existing works.

		Training					Testing			
Method	Dataset	Accuracy	2.2646	Recall	F1 Score	Time (s)	Accuracy	Precision	Recall	F1 Score
LSTM	Eye	0.9977	12.9116	0.9971	0.9976	2.2646	0.9965	0.9971	0.9956	0.9963
RexNet	Eye	0.9977	-	0.9971	0.9976	12.9116	1.0000	1.0000	1.0000	1.0000
[20] *	Eye	-	-	-	-	-	0.9845	0.9764	0.9184	0.9764
[18]	Eye	-	4.1522	-	-	-	0.8479	0.852	0.8300	0.837
LSTM	Fingernails	1.0000	144.4086	1.0000	1.0000	4.1522	0.9748	0.9771	0.9735	0.9746
RexNet	Fingernails	1.0000	-	1.0000	1.0000	144.4086	0.9958	0.9957	0.9960	0.9958
[20] *	Fingernails	-	1.9917	-	-	-	0.9833	0.9764	0.9744	0.9754
LSTM	Palm	0.9945	10.3257	0.9942	0.9945	1.9917	0.9918	0.9910	0.9926	0.9917
RexNet	Palm	0.9973	-	0.9971	0.9973	10.3257	0.9918	0.9913	0.9924	0.9918
[20] *	Palm	-	-	-	-	-	0.9912	0.9979	0.9998	0.9989
[19] **	Palm	-	Time (s)	-	-	-	0.9996	0.9979	0.9998	0.9997

* In this study, the numerical results from Tables 6–8 in [20] appear to diverge from the ROC and AUC curves shown in Figures 7–9 of the same source. The figures highlight a significant gap in AUC values, which seems to contradict the data reported in the tables. ** Similar to the observations in [20], the AUC and ROC results presented in Figures 11–13 of [19] show a noticeable difference when compared to the data in Table 5 and Table 6. Although the same data-processing methods were used, the outcomes between the proposed approach and the compared methods appear inconsistent, highlighting potential discrepancies.

**Table 5:** Results of hyperparameters tuning.

Dataset	Palmar Images		Eye Conjunctival		Fingernails	
Hyperparameter	RexNet	LSTM	RexNet	LSTM	RexNet	LSTM
Number of hidden units	61	185	109	64	59	152
Maximum epochs	19	47	48	195	17	185
Mini-batch size	163	40	89	152	161	58
Initial learning rate	0.0655	0.0128	0.0575	0.0755	0.0553	0.0969
Gradient threshold	0.9329	0.7475	0.3729	0.2756	0.8394	0.1089
L2 regularization	0.0226	0.0479	0.0051	0.0171	0.0382	0.0451
Dropout rate	0.1560	0.4716	0.4555	0.3064	0.2094	0.0548
Recurrent dropout rate	0.0550	0.4238	0.0629	0.1392	0.2459	0.2703
State activation function	tanh	tanh	soft sign	tanh	soft sign	soft sign
Gate activation function	sigmoid	sigmoid	hard sigmoid	sigmoid	sigmoid	hard sigmoid
Sequence length	80	41	15	92	12	67

**Table 6:** Networks sizes (trainable parameters).

Dataset	Algorithm	Trainable Weights
Eye	LSTM	5698
Eye	RexNet	21,802
Fingernails	LSTM	29,642
Fingernails	RexNet	12,451
Palm	LSTM	42,552
Palm	RexNet	17,875

## Data Availability

In alignment with our commitment to advancing scientific knowledge and promoting reproducibility and accessibility, the code for this study can be downloaded from: https://doi.org/10.5281/zenodo.13880127. While the data used in this work is already publicly available, as referenced within the paper, providing the code will facilitate further research, support the scientific community, and contribute to the broader impact of our findings on society and humanity.
